# Evolution of In Silico Strategies for Protein-Protein Interaction Drug Discovery

**DOI:** 10.3390/molecules23081963

**Published:** 2018-08-06

**Authors:** Stephani Joy Y. Macalino, Shaherin Basith, Nina Abigail B. Clavio, Hyerim Chang, Soosung Kang, Sun Choi

**Affiliations:** College of Pharmacy and Graduate School of Pharmaceutical Sciences, Ewha Womans University, Seoul 03760, Korea; sjymacalino@ewhain.net (S.J.Y.M.); shaherinb@gmail.com (S.B.); nabclavio@ewhain.net (N.A.B.C.); hrchang@ewhain.net (H.C.)

**Keywords:** protein-protein interaction, peptidomimetics, hot spots, network analysis, machine learning, docking, virtual screening, fragment-based design, molecular dynamics

## Abstract

The advent of advanced molecular modeling software, big data analytics, and high-speed processing units has led to the exponential evolution of modern drug discovery and better insights into complex biological processes and disease networks. This has progressively steered current research interests to understanding protein-protein interaction (PPI) systems that are related to a number of relevant diseases, such as cancer, neurological illnesses, metabolic disorders, etc. However, targeting PPIs are challenging due to their “undruggable” binding interfaces. In this review, we focus on the current obstacles that impede PPI drug discovery, and how recent discoveries and advances in in silico approaches can alleviate these barriers to expedite the search for potential leads, as shown in several exemplary studies. We will also discuss about currently available information on PPI compounds and systems, along with their usefulness in molecular modeling. Finally, we conclude by presenting the limits of in silico application in drug discovery and offer a perspective in the field of computer-aided PPI drug discovery.

## 1. Introduction

Rational drug discovery and design has progressed at a precipitous pace with the aid of in silico strategies and innovations in hardware and computational power. While still in its infancy as compared to traditional drug discovery techniques, computer-aided drug discovery (CADD) has helped deliver several success stories [1,2,3,4,5,6,7,8,9,10,11] that inspired confidence in its continuous application in the pharmaceutical industry. CADD lends cost- and labor-efficiency in the identification of potential hits for a therapeutic target before delving into extensive experimental assays. The use of computational tools requires the availability of vast amounts of information on protein and ligand structure, protein function, and an expert grasp of intermolecular forces and energies necessary for the interaction between binding partners. Its benefits encompass the different stages of drug discovery and development, including target and hit identification, structure-activity relationship studies, compound optimization, and analysis and prediction of lead pharmacokinetic properties. Nevertheless, like any other approaches, in silico methods are not infallible and are more valuable when they are employed in combination with other drug discovery tools.

In the last couple of decades, protein-protein interactions (PPIs) have become popular therapeutic targets. Proteins interact with other biologically important molecules such as peptides, proteins (homo or hetero), DNA, and RNA to carry out their functions. In general, PPIs are involved in effecting regulatory changes in response to external stimuli. Different PPI targets have been implicated in cancer, metabolic diseases, neurological disorders, and many other diseases [12,13]. The data generated through experimental techniques for these targets are often hindered in terms of manpower, cost, time, accuracy, and interactome coverage [14]. In light of this, relying only on experimental techniques may be detrimental to research efforts, as massive amounts of resources are required to fully explore the human protein interactome. Moreover, PPIs continue to be difficult targets for drug discovery efforts, due to the differences in PPI binding site properties and ligand chemical space when compared to traditional protein targets. As a solution, computational tools have been utilized to fill in the gaps, and to supplement experimental methods in the investigation of these complex targets.

In this review, we will initially discuss the significance of PPIs as therapeutic targets in the drug discovery paradigm, and how this has been dealt with so far in the research field. Next, we will expound on how various in silico methods and recent advances in this field can facilitate PPI drug discovery, as exemplified in several case studies. Lastly, the benefits and pitfalls of CADD techniques in relation to PPI drug discovery will be reviewed. Due to the breadth of PPI drug discovery, we limit the scope of this review to the computer-aided rational discovery and the development of small molecules, peptides, and peptidomimetics for PPI.

## 2. Relevance of PPIs in the Drug Discovery Paradigm

With an estimated 650,000 PPIs as part of the human interactome [15], it is evident that these interactions play crucial roles in various cellular processes and pathways. Dysregulation in PPIs are often found to be the primary cause of several disease pathologies, making them attractive drug targets. However, the development of PPI inhibitors and stabilizers have been hindered because of the seemingly low druggability of PPI interfaces. Extensive studies for PPI targets and modulators have been performed to understand these complex targets and identify distinct properties in its network, conformational structure, and ligand chemical space. PPI druggability continues to be poorly understood because these targets show substantial diversity as compared to protein families that have traditionally been targeted until now. Despite this, its relevance to multiple diseases and relative novelty in the drug discovery field encourage researchers to pursue these difficult targets. Moreover, the discovery of PPI inhibitors and therapeutics (Table 1) in the last few decades have ascertained that these targets are tractable and can be modulated by small molecule compounds.

### 2.1. Structural Features of PPIs

PPI interfaces are shallow and highly hydrophobic with large contact surfaces (>1000 Å^2^) [16,17] that were thought to be involved in their entirety for the formation of protein complexes. Due to this, PPI targets were portrayed as “intractable.” Later, alanine scanning experiments revealed the existence of “hot spots,” which are crucial residues in the protein-protein binding interface that act as chief contributors to the binding free energy. Hot spots correspond to structurally conserved regions primarily composed of tryptophan, isoleucine, arginine, and tyrosine, and often form clusters where compounds can potentially bind [18,19]. Though hot spots are frequently bundled closely together in a PPI interface, there are cases where regions are separated but still work together to allow tight attachment [20]. Aside from the presence of hot spots, other parameters, including contact surface area, polarity, flatness, and buriedness, have been employed to characterize PPI interfaces [21,22,23,24,25,26]. Generally, a PPI interface is split into a core and a rim region. The core region is buried, and consists of residues with higher hydrophobicity and conservation, whereas the rim region is in the adjacent solvent-accessible area with more polar and flexible residues [27,28,29,30]. Because the core regions are more buried and can form interfacial grooves, PPI inhibitors often target these areas and are therefore usually hydrophobic.

Different types of PPI complexes are found in biological systems. Obligate complexes are PPIs that require permanent interaction between protein partners or subunits to establish function, such as in the cases of the P22 Arc repressor [74] and V-ATPase [75]. In contrast, non-obligate PPIs have both stable functioning complexes and protomers in vivo [76], like the GroEL-GroES chaperonine system [77]. Obligate complex interfaces are usually more hydrophobic than non-obligate complex interfaces, which show higher polarities in order to exist as independent protomers in solution [17,78]. Protein partners can also bind to each other in a transient fashion, such as with key interactions involved in cell signaling and regulatory pathways. Weak transient PPIs are commonly formed by disordered proteins or oligomers that have several states in vivo, leading to interactions that are easily formed or broken based on the conformation of each protein partner. On the other hand, strong transient complexes require a molecular trigger, such as ligand binding or post-translational modification (PTM), to instigate a change in complex structure [79]. Weak transient complexes are usually mediated by changes in pH or temperature, and have small and planar surfaces, while those influenced by strong molecular triggers are often larger and more hydrophobic [80]. PPI interfaces with a surface area larger than 1000 Å^2^ are noted to be more flexible and prone to induced-fit conformational changes [17,80].

PPI complexes display a wide range of affinity and stability. However, high specificity is still observed, even with proteins that have multiple partners or “protein hubs” [81]. When dealing with binding specificity of protein hubs, key aspects include molecular recognition and binding affinity. Orientation and electrostatic complementarity between protein partners are found to be crucial for identifying the correct protein partner and forming a pre-complex. Subsequently, short range interactions between partner hot spot regions establish the binding affinity for the stabilization of the complex [82]. PPI binding interfaces exhibit various means of mediating specificity of interaction, including changes in conformation, binding site properties, and the presence of specificity-determining sites.

While hot spots are considered to be the main contributors for binding affinity and stability, not all hot spots participate in specificity, as a portion are often shared among several partners that bind to the same site of a protein hub. Separate specificity-determining hot spots that can distinguish between cognate and non-cognate partners are present in PPI binding interfaces [81,83]. The presence of anchor residues and anchoring grooves have been remarked upon as critical factors for molecular recognition between protein partners [84]. This was observed in a study done by Kimura et al. [85] wherein mutation of Lys15 of the bovine pancreatic trypsin inhibitor led to a vast decrease in association rate with trypsin. On the other hand, mutating Arg17 allowed association with trypsin but resulted in a large increase in the off rate. These indicate that although both can be classified as hot spots, each residue contributes distinctive functions in the PPI complex—Lys15 is required for recognition and initial binding, while Arg17 is critical for the stabilization of the complex [85]. For facilitated recognition and complex formation, Rajamani and colleagues [84] stated that anchor residues are usually in their bound-like conformations, while the rest of the hot regions are more flexible and buried in the unbound state. Aligned with this, molecular dynamics (MD) simulations of the receptor binding site did not exhibit any significant changes in pocket conformation or backbone rearrangements, suggesting that the grooves to which the anchor residues bind are pre-defined [84]. Interaction with several different partners can also constitute the presence of multiple binding sites, corresponding to distinct protein subunits, where various ligand proteins interact to elicit specific responses or functions [86]. Recognition of a specific protein subunit is influenced by the differences in amino acid (AA) compositions in the binding interface and the inherent diversity of PPI structural features, even among members of the same protein family [14,86].

PTM is an alternative process where proteins recognize different protein partners to carry out their specific functions. A protein can be modified using different PTMs in one or different residues at the same time, or in a sequential manner, to increase its complexity and functional scope. Data statistics from dbPTM show that over 60% of PTM sites are situated in protein functional domains involved in PPIs, implying that PTMs play a crucial role in the modulation of PPI function [87]. Phosphorylation is one of the most common type of PTMs found in cell signaling mechanisms and is frequently found on heterooligomeric and transient interfaces. It can regulate protein function by influencing specific recognition, allosteric regulation, protein modularity, and binding [88]. This is in agreement with a study conducted by Duan and Walther [89], where they noted that proteins subject to PTMs, most especially phosphorylation, are observed to have central roles and a wide interaction range in the human protein interactome [90].

Structural flexibility can also greatly affect binding affinity and specificity in PPIs. The dynamic nature of proteins allows them to alter conformations based on the required interactions for binding with a partner and inducing a corresponding function. Some proteins can subtly influence both specificity and affinity by altering the orientation of only one or a few conserved residues in the binding interface, such as MDM2 [91] and proteins with the Src homology 3 (SH3) domain [92,93]. In other cases, large global or local conformational differences are observed between different functional states. Conformational adjustment upon protein partner binding can be explained via two possible mechanisms: induced-fit [94], wherein conformational change occurs upon interaction of the partners, or population-shift [95], where it is postulated that there exists a dynamic equilibrium consisting of an ensemble of conformations from which a certain portion exhibit the conformation a particular partner preferentially binds with [95,96]. This paradigm is exhibited by intrinsically disordered proteins (IDPs) or regions (IDRs), which have high structural flexibility and diverse conformations in their native functional state. As opposed to the conventional theory, where a unique sequence defines a unique three-dimensional (3D) structure and function [97,98,99], IDPs and IDRs cover a wide range of conformational space, and have distinct structural arrangements at a given time point [97,100], enabling promiscuous and transient interactions with different protein partners. The high modularity of these structures is linked to crucial roles in cellular processes, and requires tight regulation via changes in subcellular concentration, diverse conformations, and PTMs [100,101].

For the design of efficient PPI modulators, a great level of understanding of the target is needed: the type of complex formed, elucidation of binding epitopes and hot spots, determination of binding site flexibility, etc. While more information is progressively coming to light regarding PPI binding requirements, further characterization of features that synchronize molecular recognition, affinity, and specificity within the target protein interface is vital in designing therapeutics for a specific PPI and function to improve selectivity and off-target toxicity.

### 2.2. Characteristics of PPI Modulators

Increased understanding of druggability and molecular recognition in PPIs has undoubtedly stimulated drug discovery efforts for these previously intractable targets, offering hope in identifying small molecule candidates for PPI-regulated pathways. However, the development of PPI modulators is still at a relatively slow pace in comparison to conventional small molecule drugs, due to the challenges conferred by the structural optimization required to improve the affinity and pharmacokinetic properties of the PPI ligands. The complexity of a PPI inhibitor primarily depends on the interface structure, and this is usually equivalent to that of its targets. Analysis of known PPI modulators showed that these compounds are larger, more hydrophobic, and contain more multiple bonds and aromatic rings [102,103,104]. Interestingly, PPI ligands have a higher topological polar surface area (TPSA), even with its high hydrophobicity, as compared to traditional drugs, resulting in their tendency to form more hydrogen bonds in the PPI surface. This can be attributed to their larger sizes that allow for the attachment of more polar atoms [103]. More success is acquired for PPIs with grooves or small pockets, compared to globular interfaces. However, those with hot spot pockets that are spread out across a large interface are naturally more challenging. Because of this, PPI inhibitors also tend to have a more 3D conformation than the average inhibitor [105,106], especially for extended surfaces and disjointed pockets, due to its propensity to mimic the binding epitope of the partner protein and anchor into small pockets present in PPI interfaces [104,106,107,108]. Based on these, it is evident that PPI inhibitors occupy a different chemical space as compared to typical small molecule inhibitors, and hence, should be carefully evaluated in a different manner [103,108]. Whereas the primarily hydrophobic interacting features of PPI inhibitors can be buried in hot spot regions, the rest of their structures are often solvent-exposed due to their binding location on the protein interface. These elements can be exploited to tailor pharmacokinetic properties without negatively influencing binding affinity [14].

Studies suggest that 15–40% of the human interactome is comprised of protein-peptide interactions [109]. Natural peptides have been used as leads for PPI inhibition [110,111,112] due to their biocompatibility, low toxicity, and high modularity, which can aid in both potency and selectivity [113]. However, these ligands tend to have low bioavailability because of their high propensity for proteolysis, making them poor drug candidates. Several other approaches are utilized for the discovery, design, and optimization of PPI inhibitors: (a) using unnatural AAs and other synthetic modifications to the peptide backbone for the design of peptide mimics (i.e., peptidomimetics) to improve bioactivity and pharmacokinetic properties of peptide leads; (b) exploiting natural cyclic and macrocyclic peptides that are large enough to bind to a PPI interface while still possessing favorable properties that can overcome proteolysis and difficulties in cell permeability; (c) designing and engineering miniproteins via phage display methods to target large PPI interfaces with high specificity [114,115]; (d) fragment-based drug design (FBDD) to identify low molecular weight (MW) fragments that targets hot spot clusters [116,117].

Traditionally, potency was the chief facet used for the early evaluation of lead candidates. However, it has been established that potency alone does not completely explain bioactivity against a target, and other physicochemical properties should also be considered [118]. Advances in organic and combinatorial chemistry, as well as changes in therapeutic target profiles, resulted in the growth of both chemical and drug-like spaces over the years [119,120]. Consequently, the rubrics that were often used to evaluate drug candidates before, have significantly evolved. Since PPI inhibitors are generally bigger and more hydrophobic than conventional small molecule drugs, and it is difficult to accurately assess its drug-likeness using typical metrics like Lipinski [121] and Veber rules [122]. Recent assessment of current drugs gave rise to beyond-rule of 5 (bRO5) characterization of orally bioavailable compounds [123,124]. Physicochemical properties that were noted by this novel classification, correlate well with typical PPI modulator features. Moreover, ligand efficiency (LE) and lipophilic ligand efficiency (LLE) are now more commonly used to determine drug-likeness. LE quantifies the contribution of a molecule’s structure to binding affinity, whereas LLE estimates a compound drug-likeness by correlating its potency and lipophilicity. Both measures are used to normalize potency and physicochemical properties to better assess a series of compounds [125]. The average LE for the PPI inhibitors was found to be 0.23 kcal/mol per heavy atom, while average LLE was 1.32 kcal/mol, both of which are lower compared to the preferred values of more than 0.3 [126] and 5 [127], respectively. In comparison, the average LE and LLE of a typical drug is approximately 0.45 and 4.43, respectively [118]. Aside from these, the correlation of potency with ADMET properties [118] and physicochemical properties with promiscuity or selectivity [118,127,128,129] are also currently being tackled to further expound our understanding of drug-likeness in relation to compound activity.

## 3. Emerging In Silico Approaches for PPI Drug Discovery

**Figure 1:**
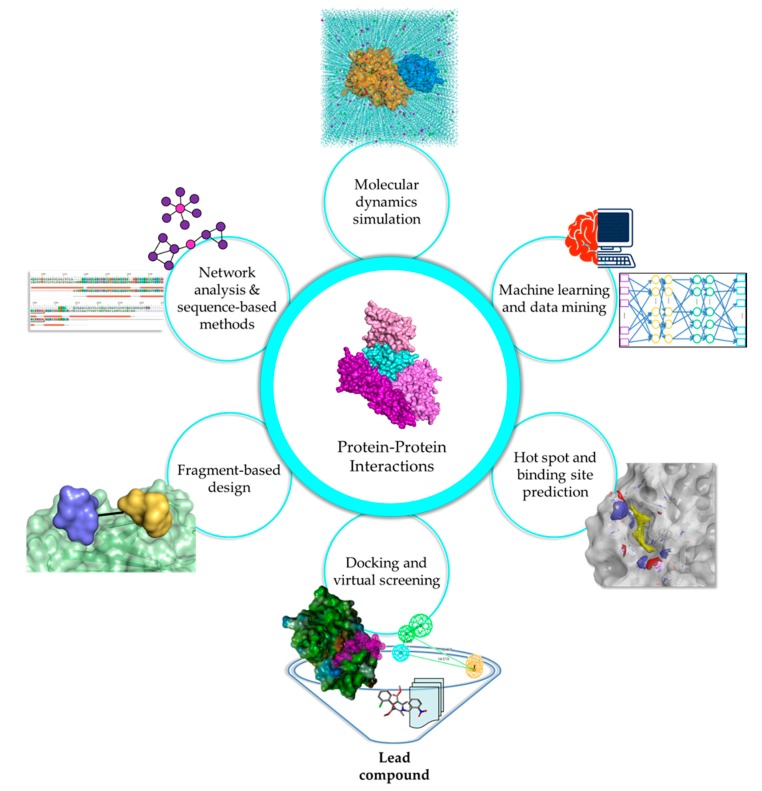
In silico strategies for PPI drug discovery. Diverse computational tools encompass various stages of PPI drug discovery, including the interpretation of protein network topology, the characterization of interface and hot spots, the exploration of PPI chemical spaces for lead discovery and optimization, and the elucidation of complex interactions and dynamics.

Experimental methods are widely accepted as the standard for any analysis as they illustrate biological scenarios in either in vitro or in vivo systems. However, the immensity of the human interactome requires considerable cost, effort, and time to study. Moreover, PPI studies are highly dependent on dynamics, PTMs, and physiological conditions, causing difficulties in distinguishing true interactions from experimental artifacts and inconsistencies in findings, especially in cases involving transient interactions and IDPs. In silico methods have emerged as alternative methods or as complements to experimental techniques, to fill in the gaps regarding vital PPI information, and to provide a foundation for further analyses (Figure 1).

### 3.1. Discerning the PPI Network Topology

PPI targets have become a class of their own throughout the years. In contrast to well-known protein families, like GPCRs and kinases, this class of targets encompasses exceedingly diverse structures and interactions. Efforts have been made to compile information about experimentally-determined PPIs into databases or platforms. Table 2 lists these repositories, which can provide invaluable information regarding functional characterization of proteins to ascertain and weigh their roles in diseases. Primary databases report only experimentally verified protein interactions from individually published studies that were manually curated to provide researchers with accessible information. Naturally, curating PPI information is no easy task; errors and noise are often found in these repositories. To ensure the robustness of study models, careful data selection must be observed by establishing replication with different methods, interaction type, subcellular location, and other physiological aspects [130,131]. Access to such extensive datasets are provided by meta-databases, which report experimentally validated PPIs collected from multiple primary databases and integrate them into one large data model.

Network analysis and sequence-based methods are valuable strategies that have widespread application in various stages of drug discovery, including target identification, binding site prediction, and polypharmacological studies [165]. Due to the impact of PPIs as therapeutic targets for diseases of interest, it is beneficial to understand the protein interactome and its correlation with disease onset and development via comprehensive mapping of the PPI network (PPIN) topology. Studying network topologies allows for the identification of novel biomarkers and relevant disease targets that would have taken a long time to discover and validate using conventional means. Subsequently, sequence and conservation information should be studied at length to determine their importance to protein dynamics and binding, further aiding in the rational design of drug candidates. The cornucopia of experimental data that is available due to advances in structural biology and biophysical methods can be expended for PPIN analysis.

PPINs exhibit typical web-like structures, wherein most proteins (nodes) display interactions (edge) with only one or a few partners, while some proteins exhibit contact with multiple partners, and are hence called hubs [166]. The correlation between topology and protein function is often measured through node degree and betweenness centrality [167,168,169]. Protein hubs exhibit a high node degree, i.e., the number of edges linked to a node, through multiple associations, rendering them as prominent elements that can affect the network function [170]. Indeed, studies have suggested that protein hubs are highly essential for cellular function, and can greatly affect the whole network when dysfunctional, making them prime drug targets [171,172,173,174]. On the other hand, proteins that display high betweenness centrality are considered as bottlenecks, and they work by regulating focal points of communication within the network [170]. Targeting bottlenecks, as compared to hubs can allow careful control of the interaction network due to their apparent impact on the strength and integrity of the whole network [175,176]. Discovery of these network components have already been established as a strategic source of novel protein targets [177,178,179].

In a recent study done by Vinayagam and colleagues [179], they performed network controllability analysis using 6339 proteins involved in 64,814 PPIs to sort proteins (nodes) as indispensable, neutral, or dispensable for the directed protein interaction network. Aside from classifying proteins, they observed that indispensable proteins are often conserved among species and enriched in signaling proteins such as kinases, while neutral proteins are often found as membrane and transcription factor proteins. Indispensable proteins were also implicated in genetic and human viral diseases, along with cancer genes. More specifically, they identified 56 genes that are most often deleted or over-expressed in nine types of cancer, with 10 genes already under investigation and 46 being proposed as new potential cancer targets. Remarkably, while indispensable nodes greatly populate current Food and Drug Administration (FDA)-approved drug targets, they found that these proteins are inadequately represented in the annotated druggable genome. They postulated that network controllability analysis would be able to aid in validating the druggable genome, as well as identify novel therapeutic targets [179]. Control theory and network analysis have been applied in other similar studies to identify essential nodes for cancer [180] and viral infection [181].

PPI prediction is essential for functional annotation. Constraints in experimental approaches necessitated the application of in silico methods, such as binding site prediction (Section 3.3) and protein-protein docking (Section 3.4). However, both these methods require prior knowledge of PPI partners and the availability of 3D structural data. In cases concerning unknown PPI partners and/or structures, sequence- and coevolution-based methods can be utilized to predict the probability of interaction and residues pertinent to binding. Multiple sequence alignment (MSA) of homologs offer knowledge about residue coevolution, which can predict protein partners based on the premise that partners usually coevolve to maintain their function [170,182]. Information obtained from these types of analyses can also facilitate the 3D structure prediction of applicable PPI complexes [183]. Another approach takes advantage of orthologous associations by predicting PPIs based on interologs, wherein proteins that are observed to interact in one species, are predicted to also interact in other species if they are found to be conserved [184]. Several machine learning (ML) models are also based on sequence information, and these have been highlighted in Section 3.2.

### 3.2. Harnessing the Power of Machine Learning Algorithms for PPIs

To extend the concept of in silico models, intelligent and contemporary ML algorithms have been implemented in the identification of PPIs. ML is a data-driven or knowledge-based approach which requires an adequate number of training sets and features. Statistical and ML methods have been applied at varied stages: assimilation of diverse heterogenous datasets, assessment of predictions, prediction of prospective PPIs, and investigation of extrapolated PPI networks [185,186,187,188].

**Figure 2:**
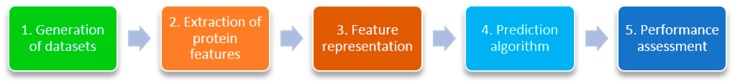
General steps involved in machine-learning (ML)-based PPI predictions.

During the past, several ML algorithms (i.e., κ-nearest neighbor (κNN), naïve Bayesian [188,189], neural networks (NN) [190], random forest (RF), support vector machines (SVM), decision trees, logistic regression, etc.) have been applied to predict PPIs. ML methods utilize a dataset of experimentally validated PPI surfaces to train interface predictors and further employ the trained model for prediction of protein-protein interfacial residues of query proteins [191]. Prevailing ML predictors apply binary classification, where a target residue is classified as either interfacial or non-interfacial by utilizing the target features or adjacent residues to formulate predictions [191]. Moreover, the accuracy of the prediction model is dependent on the input features used for training. Hence, it is crucial to identify the various protein features that are essential for training a ML algorithm. Several protein features are utilized in the development of models for PPI predictions, either individually or in combination. However, a single feature does not support sufficient data in the prediction of PPIs. Therefore, a combination of features is essential to enhance the performance of ML methods in PPI prediction. Some of the protein features utilized in model development include AA types, co-essentiality data, evolutionary information, GO-driven frequency-based similarity and semantic similarity, MIPS functional catalogue (FunCat), physicochemical properties of AAs, position-specific scoring matrices (PSSMs), protein expression data, residue interface propensity, secondary and tertiary structural information, sequence entropy, surface shape, and solvent accessible surface area [192,193]. Once the model is properly trained with the input features, its performance is assessed with an external or test dataset. The five main steps involved in PPI predictions are depicted in Figure 2. In PPI-based ML approaches, the input of prediction models is in the form of sequence or structural features or both. However, most of the existing ML interface predictors are structure-based [191]. The merits and demerits of structure- and sequence-based methods have been well reviewed in [191]. Additionally, there are a few meta-based approaches, where raw scores from several prediction servers are integrated and re-computed to improve the prediction performance. Representative structure-, sequence-, and meta-based ML predictors for identification of PPIs are summarized in Table 3.

Jones and Thornton developed a method for the prediction of PPI interaction sites by analyzing a group of residue spots on the protein interface. Each residue patch was examined using six parameters, including the accessible surface area, hydrophobicity, planarity, protrusion, residue interface propensity, and the solvation potential. The relative combined score was calculated to predict the probability of surface patch formation in the PPIs [194]. Furthermore, the distribution of the observed patch rankings relative to all other surface patches were evaluated for each dataset [195]. Likewise, Bradford et al. developed SVM- [196] and Bayesian-based [197] classifiers using surface patches. Another group developed a PPI predictor, known as SPPIDER [198], using SVM and NN in combination with relative solvent accessibility (RSA). The authors showed that the implemented RSA-based features demonstrated superior performance to other PPI-based features. Ofran and Rost developed a NN-based method, known as ISIS [190], for the prediction of protein interacting residues from sequence. This ML approach combines AA composition of protein-protein interfaces, along with evolutionary profiles, solvent accessibility, and protein secondary structural features. A naïve Bayes classifier, PSIVER, was developed by Murakami and Mizuguchi for the prediction of PPIs using sequence-based features, such as PSSM and predicted accessibility. The conditional probabilities of each AA feature were estimated using a kernel density estimation (KDE) method [199]. Two sequence-based PPI predictors, namely LORIS [200] and SPRINGS [201], were developed by applying L1-regularized logistic regression and artificial neural network (ANN) methods based on several sequential features.

Besides supervised ML algorithms, unsupervised ML approaches were also implemented in PPI prediction. Deep learning has become a new dimension of ML field, which attempts to learn multiple layered models of inputs, known as NNs. A deep learning algorithm is capable of handling extensive raw and complex data where it operates by mimicking deep neural networks (DNNs) and learning processes of the human brain. The central idea of deep learning has been detailed in [202]. It has potent applications in decision making, natural language understanding, and image and speech recognition. This algorithm has also been applied in the field of bioinformatics and biopharma industry. Recently, Sun et al. implemented deep learning for sequence-based prediction of human PPIs [203]. They applied a stacked autoencoder (SAE) to study PPIs in humans and other species (*E. coli*, *Drosophila*, and *C. elegans*). The models developed using an autocovariance (AC) coding method showed the top results on 10-fold cross validation and varied external datasets ranging from 87.99% to 99.21% (prediction accuracy). Another group developed a method known as DeepPPI, which applies DNNs to obtain high-level discriminative characteristics from common protein descriptors [204]. This method showed better performance on the external data set, demonstrating an accuracy of 92.50% and a Matthews Correlation Coefficient (MCC) of 85.08%.

ML tools can also be applied in the preparation of PPI libraries or in the analysis of initial PPI hits through different filtering routes to evaluate drug-likeness or ADME/Tox properties. Information regarding PPI modulators is available in 2P2Idb [105,228], TIMBAL [229,230], and iPPI-DB [106,231], and can be used in the development of pertinent ML-based models. A decision tree strategy, known as PPI-HitProfiler [232], was built by Reynès and colleagues based on PPI inhibitors identified in literature, and it is implemented in the FAF (Free ADME-Tox Filtering tool)-Drugs webserver [233,234,235,236]. In their method, a global physicochemical depiction of PPI inhibitors was established, along with important descriptors that relate to the molecular shape (Radial Distribution Function) and the number of multiple bonds (unsaturation index), that can be used in the classification of compounds occupying the PPI chemical space. Using different PPI complexes with ligand and bioassay information, their model was able to correctly identify 70% of the validated actives and 52% of the inactives [232]. On the other hand, Hamon et al. built an SVM tool, the 2P2I_Hunter_ [237], based on PPI modulator information taken from 2P2Idb. For the model development, they distinguished molecular descriptors pertaining to an octanol-water partition coefficient, hydrophilicity, MW, unsaturation, the number of rings, H-bond donors and acceptors, TPSA, and rotatable bonds, as being essential for categorizing the potential PPI modulators from screening libraries. Their SVM model showed a high accuracy (96%) and specificity (96%), but very low sensitivity. However, it boasted a high enrichment factor of 8, making it an efficient method for filtering out non-PPI molecules from screening libraries. Thus, the application of ML algorithm in PPI predictions provides a promising approach for the deeper understanding of the vast network of protein interactions and their modulators. However, even with the success of ML-based predictors, prediction accuracy and computational efficiency of developed models can still be improved further [238].

### 3.3. Elucidation of Interface Characteristics and Hot Spot Contribution in PPIs

The breakthrough of hot spot residue contribution to PPI binding, in conjunction with innovations in protein structure determination in the last several years, has greatly helped in characterizing protein interfaces. Even with current efforts to fully characterize the complete human interactome using well-known genetic and biochemical techniques, numerous targets are still without structures, and the sheer number of partner interactions involved leads to difficulties in fully elucidating all of them. PPIs mainly transpire in conserved regions of protein interfaces, where hotspots are usually identified, and they can cause conformational changes in the overall protein structure [239]. However, it is unwise to generalize interface topographies due to the diversity of interface features between different PPI types. Transient interactions are often found to be pharmacologically relevant, and they have garnered a great deal of interest, especially in the development of various prediction methods. However, the dynamic capability and short duration of transient complex formation makes it more challenging to experimentally characterize than permanent complexes, resulting in fewer data available for model training and development [240,241]. Transient interfaces also tend to be less conserved and exhibit different properties compared to their permanent counterparts, suggesting the need for separate predictive strategies. Protein hubs are also difficult to assess, since their binding interfaces are shared by multiple partners [242].

Alanine scanning mutagenesis (ASM) has been an indispensable tool for the identification of hot spot residues of different relevant protein targets, but this method has only been applied in a limited number of PPIs. More to the point, experimental alanine scanning entails the mutation of several residues in the interface, along with biophysical evaluation of changes in the binding energy for each mutation, making it a costly approach [243]. Computational alanine scanning is a suitable and quick substitute for hot spot detection. The PPI complex structure of interest is required to be able to estimate the change in binding energy (ΔΔG), based on the bound and unbound state of the wild type and mutated proteins [244]. This method can be combined with MD simulations, as shown first by Massova and Kollman [245]. Modifications were added in the last several years, such as the use of distance-dependent pair potentials (e.g., DrugScorePPI [246]) and interaction entropy [247] for the computation of residue-specific free binding energies, to increase the accuracy of calculation and prediction of hot spots.

Docking methods, like Optimal Docking Area (ODA) [248] and pyDockNIP module of pyDOCK [249,250], have also been used to determine interface hot spots. ODA evaluates changes in interface energies based on the residue buriedness upon the binding of protein partners, and can be applicable in characterizing both obligate and non-obligate interactions [248]. It has been used to characterize PPI complexes, including those of metallocarboxypeptidases in complex with proteinaceous inhibitors [251], and UNC-45 chaperone protein in complex with myosin [252], providing beneficial information for drug discovery efforts. Alternatively, residue normalized-interface propensity (NIP) calculated from protein-protein docking results can be employed to identify hot spot residues on the protein surface, as is applied in pyDockNIP [249].

The current rise of the big data era has aided the return and further innovation of knowledge-based techniques, especially in the context of drug discovery. The availability of sequence and structural information has allowed the exploration and identification of distinguishing features for the analysis and prediction of hot spots. Sequence-, structural-, and physicochemical-based features have been used in the development of different data-driven models and in the characterization of relevant PPIs [253]. Protein sequences can offer preliminary description and prediction of potential binding sites for relatively unstudied proteins, especially in the absence of structural information, because of the noted high conservation of interface residues [239,254]. In combination with interaction prediction mentioned in Section 3.1, coevolution-based analysis can also predict residues that are critical for binding or, when combined with statistical physics, residues that have an allosteric or indirect effect on the complex [255]. Structural homology has also been proposed as a useful metric for interface characterization, since structures evolve at a much slower pace than sequences [256] and interface structural space is well-conserved [257]. More commonly, physicochemical and 3D structural characteristics have been applied in the characterization of PPI hot spots. Previous analyses have remarked upon the opposing characteristics of the interface, wherein both high hydrophobicity and high solvent accessibility have been observed. This conflicting observation has been resolved by the identification of core and rim regions, as mentioned in Section 2.1. These physicochemical features also correlate well with distinct geometric aspects of PPI binding sites, such as residue buriedness, side chain protrusion, and surface curvature [258]. Due to the diversity exhibited by PPIs, it is difficult to rely on any individual feature for binding site and hot spot prediction, and it has repeatedly been shown that the combination of several attributes has more success in providing adequate information for PPI surfaces [259]. Knowledge-based PPI binding site and hot spot prediction algorithms are exemplified in tools like ANCHOR [260], HomPPI [261], KFC2 (Knowledge-based FADE and Contacts) [262,263,264], HotPoint [265], FTMAP [266], MINERVA [267], and PredHS [268]. These tools and more are discussed in extensive detail in other articles [244,253].

MD simulations have also been employed in the identification of druggable PPI pockets, particularly for flexible and transient systems [269]. A typical case is shown in the BCL-X_L_ protein, which exhibits drastic conformational differences between its unbound and peptide-bound structures. While BCL-X_L_ is known to be experimentally druggable [270], its apo-structure appeared to have a shallow pocket and very low druggability [271]. Analysis of its MD trajectory revealed that the BCL-X_L_ structure considerably fluctuates, resulting in the formation of a highly druggable binding region and implication that much of its druggability can be attributed to its flexible structure [271].

### 3.4. Exploring the PPI Interface through Macromolecular Docking and Virtual Screening

It has been estimated that the amount of interactions in the human interactome range from 130,000–650,000 [15,272,273,274]. However, there is a huge gap between the estimated PPIs and experimentally available structural data. Even though individual protein components are known, the determination of molecular complexes through experimental techniques is challenging, due to their transient nature. In the RCSB PDB, only limited structural data is available for protein-protein or -peptide complexes. Determination of 3D structures of protein-protein or -peptide complexes is necessary to understand their associated biological functions, to predict mutation effects, and to aid in the rational design of novel PPI-based drugs. However, due to its associated experimental difficulties along with incurred high costs and the time in determining complexes, fast and reliable computational approaches, such as macromolecular docking, is necessitated. Computational docking is one of the potent in silico tools in structure-based approaches, which has been broadly utilized for PPI-based drug discovery. This method aims to yield bound structures with low interaction modes (favorable ones) by sampling a very large number of possible binding modes and evaluating each conformation using scoring functions [191].

Elucidation of protein-protein or -peptide structural complexes through computational docking has evolved significantly due to the accumulating data on protein structures and interactions, improved energy functions, and powerful techniques to accelerate the sampling process [275]. Protein-protein or -peptide docking consists of two major stages: sampling and scoring/ranking. In the docking (sampling) stage, several conformations will be generated and, among those, only the potential ones are sampled. Sampling potential orientations for protein-protein binding using global or local searches is the first stage in a docking protocol. In a global search, the protein acting as the receptor is fixed and the other acting as the ligand is moved around the receptor, where all potential orientations between protein partners in the 3D space are explored. Though this search is computationally expensive, several docking algorithms such as Fast-Fourier transform (FFT) have been implemented to reduce docking complexity. Whereas, in a local search, the local protein features are matched to acquire a suitable complementarity. Additionally, if experimental data is available, then that information will be integrated in the sampling stage to improve the prediction accuracy of the docking results.

The docking phase can either be rigid or flexible. In rigid-body docking, there are no changes in the structural features. However, in flexible docking, conformational changes in the structures are considered [170]. The docking stage typically generates thousands of putative complexes. Hence, it is necessary to sort them and acquire the best docking solutions. In the ranking stage, conformations sampled from the first stage are rescored and ranked using several scoring functions. The fundamental principles of protein-protein docking have been well reviewed in [275]. The docking predictions can be evaluated by calculating the RMSD between the predicted and original complexes, and by measuring the ratio of the predicted interface residues to native ones. Due to the increasing number of docking algorithms developed to predict protein-protein or -peptide complexes, the Critical Assessment of Predicted Interactions (CAPRI) challenge was initiated to appraise the performance of different docking protocols [276]. Currently available protein-protein and -peptide docking tools are listed in Table 4 and Table 5, respectively.

Several studies have shown the applicability of protein-protein docking in PPI research [326,327,328]. Here, we discuss a recent study where protein-protein docking has been applied for the prediction of molecular associations between proteins. Variations in protein *S*-glutathionylation has been linked to several human diseases. It has been reported that glyoxalase II (GlxII), an antioxidant glutathione-dependent enzyme, plays a role in the *S*-glutathionylation mechanism. However, its mechanistic underpinnings remain unrevealed. In a recent study by Galeazzi et al. [329], PPIs of GlxII propensity with other cellular proteins, such as malate dehydrogenase, actin, and glyceraldehyde 3-phosphate dehydrogenase, were explored using a computational protocol involving protein-protein docking and atomistic MD simulations. The suitability of the docking programs, including ClusPro, GRAMM-X, HADDOCK, HEX, PatchDock, SymmDock, and ZDOCK was tested using a set of globular protein oligomers. A reliable protein-protein docking approach that strongly agrees with the experimental findings was developed. The docking method was applied to GlxII-protein complexes for determination of their association stabilities. Subsequently, the predicted docked complexes were subjected to MD simulations and Molecular Mechanics Poisson Boltzmann Surface Area (MM/PBSA) analysis. In silico results showed that GlxII highly interacted with actin and MDH. Therefore, this study highlighted the application of protein-protein docking approaches in the elucidation of the molecular mechanisms and thermodynamics of GlxII protein binding affinity.

Similarly, once druggability and potential binding sites in the protein interface have been established, virtual screening (VS) experiments can be performed to find potential binders that can disrupt or stabilize the target PPI. VS can be categorized as structure-based (SBVS) or ligand-based (LBVS), but the former is more useful for PPIs, since ligand information for these targets is sparse when compared to other drug targets. SB pharmacophore VS can be employed for a given target, wherein interface features are explored to generate a 3D pharmacophore model used to identify ligands with diverse scaffolds that complement the binding site and have the potential to exhibit a desired bioactivity (inhibition or stabilization) [330]. Some programs that employ the pharmacophore strategy include Catalyst [331], FLAP (Fingerprints for Ligands And Proteins) [332], HS-Pharm (Hot-Spots-guided receptor-based Pharmacophores) [333], LigandScout [334], PHASE [335], and AnchorQuery [336]. Alternatively, docking is a well-established method for SBVS, wherein ligand binding is energetically evaluated by scoring functions based on its conformation and complementarity with the binding pocket [337]. Scoring functions can be categorized as force field-, knowledge-, or empirical-based [337]. Each scoring function, in combination with docking programs, has their own strengths and weaknesses, and selecting a docking program, they should be considered carefully based on the purpose of the study, i.e., ligand binding prediction (scoring) or VS (ranking). Examples of popular protein-ligand docking programs include AutoDock [280], AutoDock Vina [338], GOLD [339], ICM [340], and Glide [341,342].

Here, we present a few case studies where VS and ligand docking protocols have been incorporated to identify small molecule PPI inhibitors or stabilizers. HIV-1 Nef is involved in infection, pathogenicity, and disease development by interacting with its own SH3 binding surface [16]. Betzi et al. [343] combined VS and high-throughput docking to identify small compounds that can bind to the HIV-1 Nef SH3 surface and prevent HIV-1 Nef/SH3 PPIs. Initially, the National Cancer Institute (NCI) diversity library was pre-filtered using 14 drug-like filters, and the identified 1420 compounds were subjected to FlexX docking protocol. The docked complexes were rescored using GFscore. The top 335 lowest energy compounds were subsequently subjected to a SH3-based pharmacophoric filter and the resulting 33 potential hits were clustered. Ten of the molecules were finally selected based on the chemical and geometrical properties for further experimental validation. Another example focuses on the Bcl-2 protein family, which are well-established targets for anticancer therapeutics due to their key roles as apoptotic regulators. Zhou et al. studied the BCL-X_L_ structure in complex with BCL-2-associated death promoter (BAD) BH3 peptide, identifying three key hydrophobic features which were used to create a pharmacophore model. To identify scaffolds with suitable pharmacological and safety profiles, they screened an in-house database of FDA-approved drugs using the generated pharmacophore model, identifying three classes of compounds with different cores. From this, they performed molecular docking on the BCL-X_L_ protein to predict and study the binding modes of Lipitor and Celexocib, both of which consist of bis-aryl substituted five-membered heterocyclic scaffold. Both compounds mimicked two out of three hydrophobic features required for interaction in the BCL-X_L_ binding pocket, leading to the design of BM-501, which showed suitable affinity to both BCL-2 and BCL-X_L_ and can function as an excellent scaffold in the design of inhibitors for the Bcl-2 protein family [344]. In a different case, Myc oncoprotein promotes cancer when it heterodimerizes with Myc-associated factor X (Max), whereas Myc homodimers suppress cancer [345]. Jian et al. utilized the available crystal structure of Max-Max homodimer to identify small compound stabilizers through VS using AutoDock. The compounds (1668) which were screened from the NCI diversity dataset were subjected to blind docking using both Max-Max homodimer and Myc-Max heterodimer structures. Subsequently, the binding sites were analyzed, and potential compounds which were specific to Max-Max homodimers were identified. These case studies are clear indications that structure-based computational approaches can be used for the discovery of small molecule modulators of PPIs.

### 3.5. Exploiting Hot Spot Regions for Fragment-Based Design

FBDD has been widely employed for different disease targets in the last several years to acquire new chemical entities, while efficiently scouring a much larger chemical space than traditional high-throughput screening (HTS) can cover. Compared to extensive compound libraries commonly used for VS, which comprise of millions of complex structures, fragment libraries often contain only hundreds to a few thousand low-MW structures [346]. Despite this, fragment screening reportedly displays higher hit rates and better LE than HTS efforts [346,347]. However, most of the current fragment libraries consist of mostly flat, aromatic structures, which can still limit the exploration of chemical space. Renewed interest for natural products due to their structural diversity and relatively safe pharmacological profiles steered the efforts to use them as a source of highly 3D fragment structures. The presence of sp3-character and stereogenicity in natural product-derived fragments can increase the chemical space explored for an FBDD study [348,349,350,351].

During optimization, fragment hits can undergo linking or growing strategies. For those found to bind in adjacent pockets in the protein interface, appropriate linkers can be selected to connect the two fragments together. On the other hand, fragments can also be grown synthetically to the neighboring pocket based on binding site analysis and rational design. Between the two, linking is found to be more difficult, since the best linkers must be identified to avoid detrimental effects on lead potency and pharmacokinetic properties. While fragment binders initially show weaker affinity in screens, their smaller size and low complexity make it easier to manage physicochemical properties during the optimization than a high-affinity HTS lead. Nevertheless, these weak binding fragments are discovered to form excellent interactions in the binding site, and thus, they contribute more than half of the favorable binding energy if the interactions are preserved during fragment-to-lead optimization [346].

While the expansive interface of PPIs is not very conducive for small molecule inhibitor discovery, the presence of multiple hot spot regions spread across the surface suggests the applicability of fragment-based methods. Distinct fragments can be identified for each hot spot, and can be later linked or grown into whole molecules with novel structures that are specifically designed for a particular PPI target [352]. FBDD has been successful in providing potential lead candidates for PPIs. As a continuation of the same study by Zhou et al. mentioned in the above section, they used the crystal structure of BCL-X_L_ in complex with ABT-737 and previous assay results to identify a fragment that could be linked to BM-501. Using part of ABT-737 as a linker, they were able to rationally design low nanomolar affinity leads for BCL-2 and BCL-X_L_. They further employed structure-activity relationship and modeling studies to improve potency and cellular activities of their compounds [344].

It is important to note that hot spot identification and analysis of the receptor protein are important elements of fragment-based design of PPI inhibitors. Pertinent fragments must be screened and bound to essential hot spot clusters, and not to negligible areas in the PPI interface. Otherwise, while tight binding compounds can be designed from suitable fragments, it may not produce the desired outcome (i.e., disruption or stabilization of PPIs). An excellent example for this is shown by Geppert and colleagues [353], where they analyzed the nuclear magnetic resonance (NMR) structures of both the apo and holo structures of IFN-α. Potential binding cavities were identified using PocketPicker [354], and interface hot spots were predicted using iPred [355]. Remarkably, hot spot prediction was in good agreement with previous mutation studies and was able to identify the major interaction groove in the protein interface. Pharmacophore generation and VS of commercially available fragment-sized compounds was performed, resulting in the identification of six binders. From these, the highest-ranking compound displayed good affinity, despite its low molecular weight (279 Da). Probing the ligand protein hot spots can also facilitate the design of PPI inhibitors. The peptide backbone appropriately positions hot spot residues to surface pockets, which are primarily composed of hydrophobic groups, while forming a number of hydrogen bonds in other parts of the interface, such as in the cases of p53-MDM2 and XIAP-caspase 9 complexes. In this event, it is beneficial both to find fragments that can bind to the surface hot spot regions, and to find linkers that can fulfill the hydrogen bond requirements for interaction with the receptor protein [356].

### 3.6. Unraveling the Structural and Functional Aspects of PPIs Using MD Simulations

Despite steady progress in the growth of PPI data, comprehensive understanding of such complexes and their dynamics remains inadequate. The scarcity of 3D structural data for protein-protein or -peptide interacting complexes delays PPI research. Moreover, crystallography cannot detect the presence of druggable hot spots and transient pockets on protein-protein interfaces, due to their active motions. MD simulations surpass these limitations by facilitating in-depth analysis of structural, functional, and dynamic aspects of PPI models. They also provide valuable insights into PPI mechanisms which could be utilized in the design of PPI modulators.

**Figure 3:**
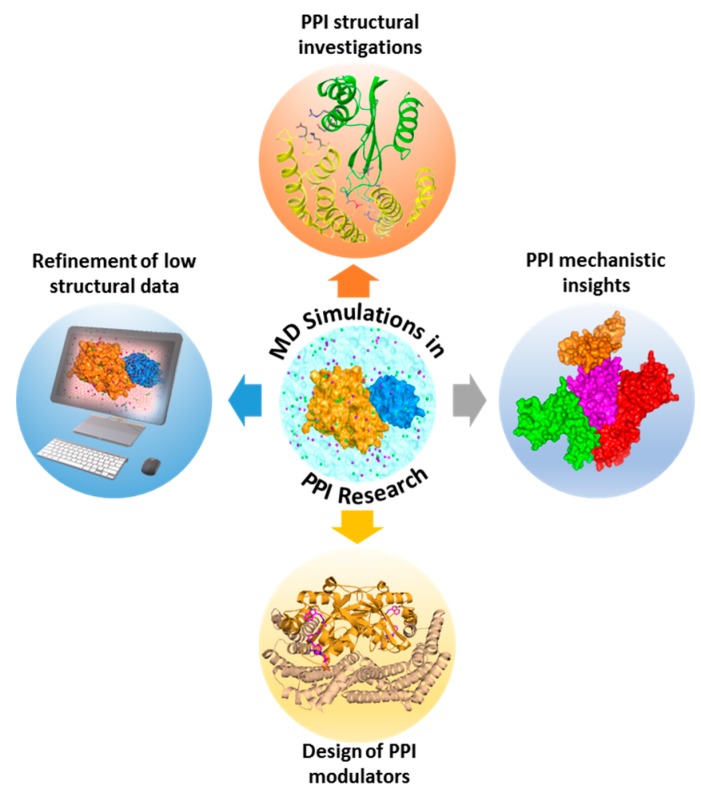
Varied applications of molecular dynamics (MD) simulations in PPI research. Using MD simulations, several aspects of PPIs can be explored, such as gaining insights into their structural, functional, and mechanistic processes, design of PPI inhibitors or stabilizers, and refinement of PPI structures with lower resolutions.

MD simulation is a commonly applied in silico approach for calculating the time-dependent motion of biological molecules. The initial step in MD simulation of a PPI is the acquisition of a starting complex structure, which could be obtained through experiments (X-ray, NMR, or cryo-electron microscopy (EM)), modeling approaches (homology modeling or protein-protein docking), or PPI databases. Once the structural template is available, the next step is to prepare the system and subject it to atomistic simulations in line with software-dependent simulation procedures. Firstly, the system’s initial positions and velocities are fixed. Subsequently, the forces among all atoms are defined by using a preconfined interaction potential, where time-dependent motions of a system could be traced by solving classical equations of motions [357]. Key utilities of MD simulations in PPI exploration include structural investigations of PPIs (e.g., identification of hot spots, functional mechanisms of complexes, possible binding partners, key interacting features of complexes, and oligomerization mechanistic insights), design of PPI modulators, elucidation of macromolecular mechanisms, and refinement of low-resolution structural data (Figure 3). In the case studies below, we highlight the benefits of MD to PPI investigations.

Dixit et al. investigated the functional mechanism of heat-shock protein (Hsp) 90 using atomistic MD simulations, along with the modeling of principal correlated motions and energy landscape analysis [358]. Through this analysis, several key areas in the structure-functional depiction of controlled interactions and the center of the communication networks were identified. In another study, Ozdemir et al. unraveled the atomistic interactions of the Rho GTPases, Cdc42 and Rac1, with the scaffolding protein IQ motif-containing GTPase-activating protein 2 (IQGAP2) using all-atom MD simulations, site-directed mutagenesis, and Western blotting [359]. They were able to decipher the underlying mechanism of the different stoichiometries involved in the binding of Rac1 and Cdc42 to GRD, and IQGAP2 dimerization. It also provided insights on how Cdc42 and Rac1 mediate actin polymerization in metastasis. The outcomes of these studies assist in the design of inhibitors and additionally provide structural, stoichiometric, and working insights into the allosteric control of the complex and protein machinery dynamics.

MD simulations can also assist in PPI hot spot prediction to provide clues for the design of potential PPI inhibitors. Survivin protein is found to be over-expressed in several solid tumors; hence disruption of its interaction with substrate proteins is indispensable for the treatment of cancer. Sarvagalla et al. [360] provided an exhaustive atomistic detail of the dimer interface of survivin-chromosomal passenger complex (CPC) protein by applying a knowledge-based model for identification of hot spot residues. Subsequently, extensive MD simulations were utilized to produce an ensemble of conformations which were further utilized for the estimation of binding free energies of identified hot spots using MM/PBSA and per-residue energy decomposition. Survivin and CPC interface hot spots were identified from these analyses. Finally, a pharmacophore model based on the hot spots was generated and virtually screened against compound databases for the identification of a potential inhibitor targeting survivin-CPC interaction. Besides hot spot predictions and functional characterization of PPIs, MD simulations could be utilized for identification of plausible binding partners. It is hypothesized that Psalmopeotoxin I (PcFK1) targets subtilisin-like serine protease, PfSUB1. To investigate this hypothesis, Bastianelli et al. [361] applied bioinformatics analysis, protein-protein docking, MD simulations, and free energy calculations on these two proteins. MD simulations was utilized for refining the docked protein-protein complexes and free energy calculations. Their computational results were confirmed via experimental testing on PfSUB1 purified and active recombinant enzyme, leading to the validation that PcFK1 indeed targets PfSUB1 enzymatic activity.

It has been widely accepted that most proteins function as oligomers. However, the oligomerization process often remains unclear. In such scenarios, MD simulations can assist experimentalists in unraveling the oligomerization processes. Zhang et al. investigated the formation of small oligomers in the amyloid fibril-formation process of the peptide GNNQQNY from the yeast prion-like protein Sup35, using explicit solvent MD simulations. Their results demonstrated that primarily antiparallel dimer forms, and subsequently novel peptides may complement the assemblies in parallel arrangement, which is in line with the experimental microcrystal structure of the amyloid fibril [362]. In another recent study, interactions between Aβ_1–42_ oligomers with each of the four models of the full-length Amylin_1–37_ oligomers were explored using extensive MD simulations, resulting in the elucidation of the link between type 2 diabetes and Alzheimer’s disease [363]. Thus, MD simulation stands as an indispensable tool to complement experimental screening techniques in PPI research that dissect PPIs at an atomistic level, with its high accuracy, vigorous validation of force fields, and the extensive availability of computational resources for performing large-scale simulations of complex protein systems.

## 4. Pitfalls of CADD in the Discovery of PPI Inhibitors

There is no doubt that computational methods have become one of the cornerstones of drug discovery, especially with the advances in technology and the increase in experimental data in the last several years. However, as with any other drug discovery and design methods, CADD has its own strengths and weaknesses when dealing with a variety of drug targets, including PPIs.

Prediction and filtering tools are rapid and dependable strategies, particularly for targets with insufficient information at the start of a drug discovery effort. In the absence of 3D information, sequence-based methods can provide quick and useful insights about conservation and evolution information, which may also be critical for ligand design. However, information that is taken from these techniques might not translate well into 3D systems [253]. In fact, Keskin et al. remarked that conservation-based analysis is not applicable to transient interactions due to its poor conservation across species [170]. Most computational tools are dependent on the availability of structural information, such as in the case of energy- and knowledge-based techniques. Energy-based methods are built on the understanding of energy terms in complex systems. Due to the intricacy of protein structures, dynamics, and interactions, several approximations are frequently included in the development of these methods to create a balance between speed and accuracy; thus, they are more useful as estimates for prioritization rather than data that is convertible to real scenarios [364]. On the other hand, knowledge-based methods make use of currently available protein and ligand information, and models created from this technique are only as good as the data used to create it. For example, certain descriptors are more predictive for specific PPI types [253]. Furthermore, if a model is based on a particular protein family or ligand class, it cannot be reliably employed to characterize other protein families or ligand classes. This becomes a hindrance with respect to the intrinsic flexibility and diversity of PPIs.

FBDD is notably more efficient than conventional HTS, both in experimental and virtual form. However, several things are needed before pursuing this strategy. First, structural information is required for fragment screening, making it an unsuitable method for relatively unknown targets. Furthermore, information about hot spot regions is crucial, as these are the focus for any type of screening efforts. Second, binding affinity and orientation of fragments may differ from that of the complete molecule. Linker groups and attachment points must be considered conscientiously, as their addition to identified fragment hits can greatly affect both the potency and pharmacokinetic properties of the lead. Third, and the same as other computational strategies, virtual fragment hits must be validated. However, validation for these low affinity structures often requires more sensitive and sophisticated methods and expertise, as compared to conventional HTS [346].

Perhaps the most notable challenge for many computational methods is the dynamic nature of proteins, as the inclusion of flexibility is extremely computationally expensive, leading to the use of rigid protein structures by the majority of CADD modules. This drawback becomes more prominent in PPIs, whose conspicuously flat interfaces are postulated to be highly flexible. MD simulations assuages these concerns as it can approximate protein movements in a carefully monitored system. Improvements in hardware computational power have also aided increased interest and utilization of MD for the analysis of PPIs [271,365,366].

The listed shortcomings for each method should not be taken as a dissuasion of their use. In fact, this is a reinforcement that more studies are needed to further improve current computational tools. Moreover, the strengths of each method can be used to make up for the weaknesses of the others. The combination of two or more CADD tools, along with the incorporation of experimental techniques, can result in better insights and success in drug discovery endeavors.5. Conclusion and Prospects of PPI Drug Discovery.

PPIs have become prime drug targets due to their integral roles in cell signaling and regulatory pathways. While increased interest for these targets encouraged comprehensive studies about PPINs, interface conformations and interactions, and PPI modulators, much is still needed to completely characterize this vast system and to overcome hindrances associated with PPI drug discovery. Current experimental techniques have facilitated our growing understanding of PPIs, resulting in the construction of informative databases containing valuable information involving the human proteome and interactome. Unfortunately, the immensity of the human interactome renders experimental methods insufficient, necessitating the use of their computational counterparts. CADD impacts various stages of PPI drug discovery by assisting in the characterization of PPIs and its unique chemical space, thereby providing better insights into the design of PPI modulators. Innovations in computational power and algorithms, along with the growing knowledge and better parameterization of macromolecular dynamics and energetics relating to PPIs, have led to notable contributions of in silico methods in various PPI drug discovery efforts.

Overall, we can expect a remarkable future for PPI drug discovery as both in silico and experimental methods continue to evolve. While each approach has their own significance in the field of drug discovery and development, it is important to remember that one is not designed to outshine the other, but to complement it. The integration of different tools, especially in the study of diverse drug targets such as PPIs, can enhance our understanding of these targets and aid in the design of potent and effective PPI modulators.

## Figures and Tables

**Table 1:** Examples of protein-protein interaction (PPI) modulators in clinical trials or clinical use.

Compound	Structure	Mode of Action	Identification Method	Clinical Status	Ref.
**ABT-199 (Venetoclax)**	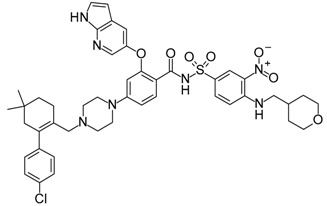	Bcl-2/(BAX/BAK) inhibitor	Rational design for BCL-2	Approved for chronic lymphocytic leukemia (CLL) with 17p deletion	[31]
**ABT-263 (Navitoclax)**	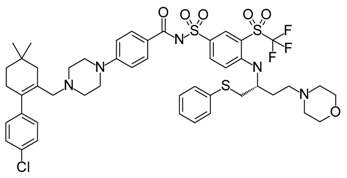	Bcl-2/(BAX/BAK) inhibitor	High-throughput screening (HTS) and fragment-based design	Phase 1/2 for various cancer types	[32,33,34,35]
**AMG232**	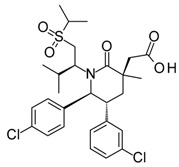	p53/MDM2 inhibitor	Fragment-based design	Phase 1 for cancer	[36]
**Birabresib (OTX015)**	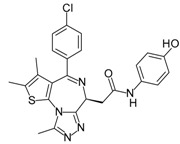	Bromodomain and extra-terminal (BET)/histone peptide inhibitor	Cell assays	Phase 1 for various cancer types	[37,38]
**Birinapant (TL32711)**	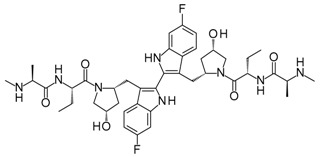	CIAP1-BIR3/Caspase-9 and XIAP-BIR3/second mitochondrial activator of caspases (SMAC) inhibitor	Dimerized SMAC mimetics	Phase 1/2 for various cancer types	[39,40,41,42]
**Cabazitaxel**	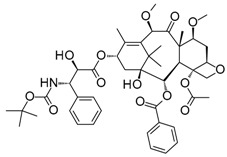	Microtubule inhibitor	Screening of semisynthetic taxane derivatives	Approved for prostate cancer	[43]
**CGM097**	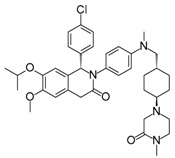	p53/MDM2 inhibitor	Virtual screening (VS), molecular modeling, and rational design based on crystal complex structure	Phase 1 for cancer	[44]
**Cilengitide**	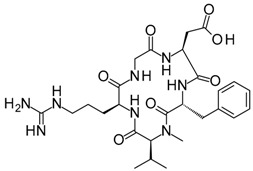	Integrin αvβ3/αvβ5 inhibitor	Ligand-based design using Arg-Gly-Asp (RGD)-binding motif	Phase 1/2/3 for various cancer types	[45,46,47,48]
**CPI-0610**	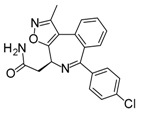	BET/histone peptide inhibitor	Structure-based drug design	Phase 1/2 for various cancer types	[49,50,51]
**Docetaxel**	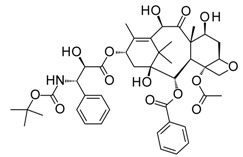	Microtubule inhibitor	Semisynthetic taxane derivative	Approved for various cancer types	[52,53,54,55]
**DS-3032b**	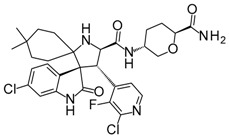	p53/MDM2 inhibitor	Enzyme and cell assays	Phase 1 for leukemia	[56]
**Eptifibatide**	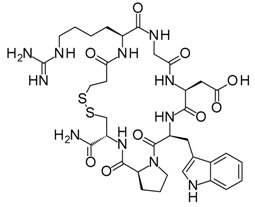	Glycoprotein IIb/IIIa inhibitor	Peptide-based (barbourin) design	Approved as platelet aggregation inhibitor	[57,58]
**FK506 (Tacrolimus)**	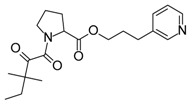	FK506-binding protein 12 (FKBP12)/Calcineurin inhibitor	In vitro and in vivo assays	Approved for immunosuppression/organ rejection	[59]
**I-BET762 (Molibresib)**	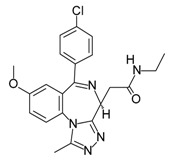	BET/histone peptide inhibitor	Cell-based HTS	Phase 2 for cancer	[60]
**LCL161**	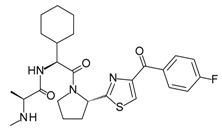	Inhibitor of apoptosis (IAP)/SMAC inhibitor	SMAC mimetics, cell assays	Phase 1/2 for various cancer types	[61,62,63,64].
**Lifitegrast (SAR1118)**	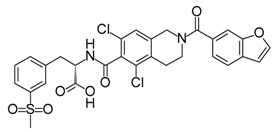	Lymphocyte function-associated antigen-1 (LFA-1)/Intercellular adhesion molecule 1 ICAM-1 inhibitor	Structure-based rational design based on LFA-1 and ICAM-1 binding	Approved for dry eye	[65]
**MI-77301 (SAR405838)**	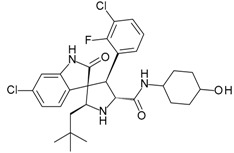	p53/MDM2 inhibitor	Structure-based rational design based on p53 peptide	Phase 1 for cancer	[66,67]
**Maraviroc**	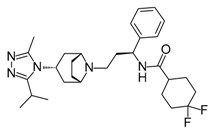	CCR5/gp120 inhibitor	HTS	Approved for human immunodeficiency virus (HIV)	[68,69]
**Onalespib (AT13387)**	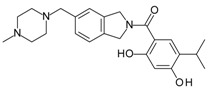	HSP90 inhibitor	Fragment-based design	Phase 1 for cancer	[70]
**RG7112**	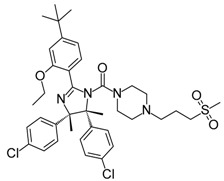	p53/MDM2 inhibitor	HTS and rational optimization of Nutlins	Phase 1 for cancer	[71]
**RG7388 (Idasanutlin)**	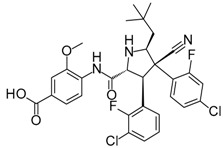	p53/MDM2 inhibitor	Rational optimization of RG7112, biochemical and cell assays	Phase 3 for acute myeloid leukemia, phase 1/2 for other various cancer types	[72]
**Tirofiban**	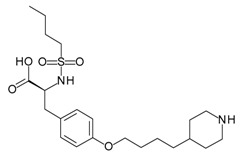	Glycoprotein IIb/IIIa inhibitor	Ligand-based design using RGD-binding motif	Approved as platelet aggregation inhibitor	[57,73]

**Table 2:** Primary databases and meta-databases * for PPI information.

Database	Website	Description	Number of Proteins	Number of Interactions	Ref.
BIND	http://download.baderlab.org/BINDTranslation/	Biomolecular Interaction Network Database (Last update: 2004)	-	198,905	[132]
BioGRID	https://thebiogrid.org/	Biological General Repository for Interaction Datasets (Last update: 2018)	-	1,202,227	[133,134]
DIP	http://dip.doe-mbi.ucla.edu/dip/	Database of Interacting Proteins (Last update: 2017)	28,823	81,762	[135]
HPRD	http://www.hprd.org/	Human Protein Reference Database (Last update: 2009)	30,047	41,327	[136]
IntAct	https://www.ebi.ac.uk/intact/	IntAct Molecular Interaction Database (Last update: 2013)	105,180	805,177	[137,138,139]
MINT	https://mint.bio.uniroma2.it/	Molecular INTeraction database (Last update: 2013)	25,178	123,940	[140]
MIPS	http://mips.helmholtz-muenchen.de/proj/ppi/	Mammalian Protein-Protein Database (Last update: 2005)	900	1800	[141]
CORUM	http://mips.helmholtz-muenchen.de/corum/	Comprehensive resource of mammalian protein complexes	-	2837	[142,143]
DroID	http://www.droidb.org/Index.jsp	Drosophila Interactions Database (Last update: 2017)	-	262,631	[144,145]
APID *	http://cicblade.dep.usal.es:8080/APID/init.action	Agile Protein Interactomes Dataserver	90,379	678,441	[146]
HIV Interaction DB *	https://www.ncbi.nlm.nih.gov/genome/viruses/retroviruses/hiv-1/interactions/	Interactions between HIV-1 proteins with host cell proteins, other HIV-1 proteins, or proteins from HIV-associated disease organisms	-	2589	[147,148,149,150]
HPID *	http://wilab.inha.ac.kr/hpid/	Human Protein Interaction Database	4642	719,349	[151]
HPIDB *	http://hpidb.igbb.msstate.edu/index.html	Database for host-pathogen interactions (Last update: 2016)	-	45,238	[152,153]
IRefWeb *	http://wodaklab.org/iRefWeb/	Consolidated protein interaction database with provenance	66,701	1,119,604 (distinct: 263,479)	[154,155]
MatrixDB *	http://matrixdb.univ-lyon1.fr/	Extracellular Matrix Interaction Database	-	9262	[156,157,158]
Mentha *	http://mentha. uniroma2.it/	Molecular interaction database (Last update: 2018)	89,666	707,003	[159]
PDZBase *	http://abc.med. cornell.edu/pdzbase	Database of PPIs which involve PDZ domains	-	~300	[160]
PICKLE *	http://www.pickle.gr/	Protein InteraCtion KnowLedgebasE	-	120,882	[161,162]
PINA *	http://omics.bjcancer.org/pina/	Protein Interaction Network Analysis	-	365,930	[163,164]

**Table 3:** Alphabetical listing of machine learning predictors for the identification of PPIs.

Tool/Server	Input Type	ML Algorithm	Features	Website URL	Ref.
Bock et al.	Structure	Support vector machine (SVM)	Primary structure and associated data	N/A	[205]
Chen et al.	Structure	Decision tree	Domain interaction data	Source code available upon request	[206]
Cons-PPISP	Structure	Neural network (NN)	Position-specific scoring matrix (PSSM), solvent accessibilities, and spatial neighbors of each residue	http://pipe.scs.fsu.edu/ppisp.html	[207]
CPORT *	Structure-based meta server	Scoring function	Combines six interface prediction methods: WHISCY, PIER, ProMate, cons-PPISP, SPPIDER, and PINUP into a consensus predictor	http://haddock.science.uu.nl/services/CPORT/	[208]
DeepPPI	Sequence	Deep neural network (DNN)	Sequence features	http://ailab.ahu.edu.cn:8087/DeepPPI/index.html	[204]
Dohkan et al.	Structure	SVM	Domains and amino acid compositions	N/A	[209]
InterProSurf	Structure	Scoring function	Solvent accessible surface area (SASA), propensity of interface residues	http://curie.utmb.edu/prosurf.html	[210]
MetaPPI *	Structure	Scoring function	Raw scores from five prediction servers PPI−Pred, PPISP, PINUP, Promate, and SPPIDER	http://projects.biotec.tu-dresden.de/metappi/	
Meta-PPISP *	Structure	Linear regression	Raw scores from three other servers: ProMate, PINUP, cons-PPISP	http://pipe.scs.fsu.edu/meta-ppisp.html	[211]
PAIRpred	Sequence or structure	Multiple pairwise kernel SVMs	Structural features: relative accessible surface area (rASA), residue depth, half sphere amino acid composition, protrusion index. Sequence features: PSSM and predicted rASA	Python code available at: http://combi.cs.colostate.edu/supplements/pairpred/	[212]
PIER	Structure	Partial least square (PLS) regression	Solvent accessibility and evolutionary conservation	http://abagyan.ucsd.edu/PIER/	[213]
PINUP	Structure	Empirical energy function	Side-chain energy score, residue interface propensity, and residue conservation score	http://sysbio.unl.edu/services/PINUP/	[214]
PPiPP	Sequence	NN	Binary encoding of 20 amino acids and PSSM	http://mizuguchilab.org/netasa/ppipp/	[215]
PPI_SVM	Structure	SVM	Physical interactions of constituent domains	N/A	[216]
Pred-PPI	Sequence	SVM	Conservation, electrostatic potential, hydrophobicity, propensity of interface residues, surface shape, and solvent accessible surface area	http://cic.scu.edu.cn/bioinformatics/predict_ppi/	[217]
predPPIS	Sequence	SVM and Bayesian classifiers	Sequence features	http://bsaltools.ym.edu.tw/predppis/	[218]
PresCont	Structure	SVM	SASA, hydrophobicity, conservation and the local environment of each amino acid on the protein surface	http://bioinf.ur.de/php/prescont.ph	[219]
PredUs	Structure	SVM	SASA, hydrophobicity, conservation and the local environment of each amino acid on the protein surface	http://bhapp.c2b2.columbia.edu/PredUs/	[220]
PRISM	Structure	Scoring function	Geometric complementarity, conservation	http://cosbi.ku.edu.tr/prism/index.php	[221]
PROFisis	Sequence	NN	Sequence features	http://rostlab.org/owiki/index.php/PROFisis	[190]
ProMate	Structure	Composite probability	Multiple features like amino-acid propensities, pairwise amino-acid distribution, residue conservation, geometric properties, etc.	http://bioinfo41.weizmann.ac.il/promate/promate.html	[222]
ProPrInt	Sequence	SVM	Sequence features, PSSM	http://crdd.osdd.net/raghava/proprint/	[223]
PSIVER	Sequence	Naïve Bayes classifier	PSSM, predicted solvent accessibility	http://mizuguchilab.org/PSIVER/	[199]
SHARP2	Structure	Scoring function	Solvation potential, hydrophobicity, accessible surface area, residue interface propensity, planarity and protrusion	N/A	[224]
SPPIDER	Sequence	SVM, NN	Fingerprints of protein interactions based on predicted relative solvent accessibility (experimental)	http://sppider.cchmc.org/	[198]
Sun et al.	Sequence	DNN	Sequence features	N/A	[203]
UNISPPI	Sequence	Decision tree	Amino acid frequencies	N/A	[225]
WHISCY	Structure and multiple sequence alignment (MSA)	Scoring function	Residue conservation, interface propensity of residues	http://milou.science.uu.nl/services/WHISCY/	[226]
Yan et al.	Sequence	SVM, Bayes	Interface residue neighborhoods	N/A	[227]

* Meta-based ML predictors of PPIs.

**Table 4:** Alphabetical listing of available protein-protein docking tools.

Tool/Server	Sampling Algorithm	Website URL	Type	Ref.
3D-Garden	Marching cubes algorithm	http://www.sbg.bio.ic.ac.uk/~3dgarden/	Online	[277]
ATTRACT	Normal-mode analysis (NMA)	www.attract.ph.tum.de	Online	[278]
ASPDock	Fast Fourier transform (FFT)	http://biophy.hust.edu.cn/ASPDOCK.html	Online	[279]
AutoDock	Genetic algorithm (GA)	http://autodock.scripps.edu/	Standalone	[280]
BiGGER	FFT	N/A	Standalone	[281]
Cell-Dock	FFT	http://mmb.pcb.ub.es/~cpons/Cell-Dock/	Standalone	[282]
ClusPro	FFT	https://cluspro.org	Online	[283]
DOCK/PIERR	FFT	http://clsb.ices.utexas.edu/web/dock.html	Online	[284]
DOT	FFT	http://www.sdsc.edu/CCMS/DOT/	Standalone	[285]
ESCHER NG	NSC algorithm	http://www.ddl.unimi.it/escherng/index.htm	Standalone	[286]
F2Dock	FFT	http://www.cs.utexas.edu/%7Ebajaj/cvc/software/f2dock.shtml	Online upon request	[287]
FiberDock	NMA	https://bioinfo3d.cs.tau.ac.il/FiberDock/	Online	[288]
FireDock	Monte-Carlo (MC)	http://bioinfo3d.cs.tau.ac.il/FireDock/	Online	[289]
FRODOCK	FFT	http://frodock.chaconlab.org/	Online	[290]
FTDock	FFT	http://www.sbg.bio.ic.ac.uk/docking/ftdock.html	Standalone	[291]
GalaxyPPDock	Cluster-guided Conformational space annealing (CG-CSA)	http://seoklab.github.io/GalaxyPPDock/	Standalone	N/A
GAPDOCK	GA	N/A	Standalone	[292]
GRAMM	FFT	http://vakser.compbio.ku.edu/main/resources_gramm.php	Online or standalone	[293]
HADDOCK	Simulated annealing	http://www.bonvinlab.org/software/haddock2.2/	Online or standalone	[294]
HDOCK	FFT	http://hdock.phys.hust.edu.cn/	Online	[295]
Hex	Spherical polar Fourier correlations	http://hexserver.loria.fr/	Online or standalone	[296]
ICM-DISCO	MC	http://www.molsoft.com/index.html	Standalone	[297]
ICM-Pro	MC	http://www.molsoft.com/icm_pro.html	Standalone	[297]
InterEVDock	FFT	http://mobyle.rpbs.univ-paris-diderot.fr/cgi-bin/portal.py#forms::InterEvDock2	Online	[298]
LightDock	Glowworm Swarm optimization	https://life.bsc.es/pid/lightdock/	Standalone	[299]
LZerD	Geometric hashing	http://kiharalab.org/proteindocking/lzerd.php	Standalone	[300]
MEGADOCK	FFT	http://www.bi.cs.titech.ac.jp/megadock/	Standalone	[301]
MolFit	FFT	http://www.weizmann.ac.il/Chemical_Research_Support/molfit/	Standalone	[302]
PatchDock	Geometric hashing	http://bioinfo3d.cs.tau.ac.il/PatchDock/	Online	[303]
PEPSI-Dock	FFT	https://team.inria.fr/nano-d/software/PEPSI-Dock/	Standalone	[304]
PIPER	FFT	https://www.schrodinger.com/piper	Standalone	[305]
PI-LZerD	Geometric hashing	http://kiharalab.org/proteindocking/pilzerd.php	Standalone	[300]
PROBE	MC	http://pallab.serc.iisc.ernet.in/probe/	Online	[306]
PRUNE	MC	http://pallab.serc.iisc.ernet.in/prune/	Online	[306]
pyDock	FFT	https://life.bsc.es/pid/pydock/	Standalone	[250]
pyDockWEB	FFT	https://life.bsc.es/pid/pydockweb	Online	[307]
RosettaDock	MC	http://rosie.rosettacommons.org/docking2/submit	Online	[308]
SKE-DOCK	Geometric hashing	http://www.pharm.kitasato-u.ac.jp/bmd/files/SKE_DOCK.html	Online upon request	[309]
SmoothDOCK	FFT	http://smoothdock.ccbb.pitt.edu/	Online	[310]
SwarmDock	NMA	https://bmm.crick.ac.uk/~svc-bmm-swarmdock/	Online	[311]
SymmDock	Geometric hashing	http://bioinfo3d.cs.tau.ac.il/SymmDock/	Online	[303]
UDOCK	MC	http://udock.fr/	Standalone	[312]
ZDOCK	FFT	https://zlab.umassmed.edu/zdock/http://zdock.umassmed.edu/	Standalone Online	[313]

**Table 5:** Alphabetical listing of available protein-peptide docking tools.

Tool/Server	Docking Algorithm	Website URL	Type	Ref.
AnchorDock	Global peptide docking	N/A	Standalone	[314]
CABS-dock	Global peptide docking	http://biocomp.chem.uw.edu.pl/CABSdock	Online	[315]
DINC	Global peptide docking	http://dinc.kavrakilab.org/	Online	[316]
FlexPepDock	Local peptide docking	http://flexpepdock.furmanlab.cs.huji.ac.il/	Online	[317]
GalaxyPepDock	Local peptide docking	http://galaxy.seoklab.org/cgi-bin/submit.cgi?type=PEPDOCK	Online	[318]
HADDOCK peptide	Local peptide docking	http://www.bonvinlab.org/software/haddock2.2/	Standalone, online	[319]
HPEPDOCK	Global peptide docking	http://huanglab.phys.hust.edu.cn/hpepdock/	Online	[320]
MDockPep	Global peptide docking	N/A	Standalone	[321]
pepATTRACT	Global peptide docking	http://mobyle.rpbs.univ-paris-diderot.fr/cgi-bin/portal.py#forms::pepATTRACT	Online, standalone	[322]
PepCrawler	Local peptide docking	http://bioinfo3d.cs.tau.ac.il/PepCrawler/	Online, standalone	[323]
PepSite	Local peptide docking	http://pepsite2.russelllab.org/	Online	[324]
PEP-SiteFinder	Local peptide docking	http://bioserv.rpbs.univ-paris-diderot.fr/services/PEP-SiteFinder/	Online	[325]
